# Cervical Ganglioneuroma in Neurofibromatosis Type 1 Presenting as a Carotid Space Mass With Postoperative Horner Syndrome

**DOI:** 10.7759/cureus.113079

**Published:** 2026-07-21

**Authors:** Rebekah Chetcuti, Bernard Galea

**Affiliations:** 1 Surgery, Mater Dei Hospital, Msida, MLT; 2 Otolaryngology - Head and Neck Surgery, Mater Dei Hospital, Msida, MLT

**Keywords:** carotid space mass, cervical ganglioneuroma, ganglioneuroma, horner’s syndrome, neck mass, neurofibromatosis type 1 (nf-1), neurogenic tumour, postoperative horner syndrome, sympathetic chain

## Abstract

Ganglioneuromas are uncommon benign tumours derived from neural crest cells and composed of mature ganglion and Schwannian stromal elements. They most frequently arise in the posterior mediastinum and retroperitoneum, while cervical involvement is rare. Although ganglioneuroma may occur in association with neurofibromatosis type 1, such presentations are infrequently reported. Surgical excision is generally curative; however, postoperative sympathetic dysfunction, including Horner syndrome, may occur because of the close anatomical relationship of these tumours to the cervical sympathetic chain.

We report the case of a 24-year-old woman with sporadic neurofibromatosis type 1 who presented with right-sided neck pain and temporomandibular discomfort. Clinical examination revealed a firm, non-tender, non-pulsatile mass in the right anterior cervical triangle, with preserved vocal cord mobility on flexible nasoendoscopy. Magnetic resonance imaging demonstrated a well-circumscribed fusiform lesion within the right carotid space extending from C1 to C6, with anterolateral displacement of the common carotid artery and splaying of the carotid bifurcation, consistent with a neurogenic tumour. Following multidisciplinary discussion, the lesion was excised via a transcervical approach. Histopathological examination confirmed ganglioneuroma with degenerative changes and no malignant features.

Postoperatively, the patient developed ipsilateral ptosis and miosis consistent with Horner syndrome. Ophthalmological assessment confirmed sympathetic dysfunction. Her symptoms gradually resolved, with complete recovery by six months. Follow-up magnetic resonance imaging demonstrated no residual or recurrent disease, and serial imaging over four years showed no recurrence and stability of a smaller contralateral lesion.

This case highlights cervical ganglioneuroma as a rare differential diagnosis for carotid space masses in patients with neurofibromatosis type 1. Although complete excision offers an excellent prognosis, careful preoperative counselling and operative planning are essential because of the risk of temporary sympathetic chain dysfunction, particularly in lesions extending toward the skull base.

## Introduction

Ganglioneuromas are uncommon, benign, well-differentiated tumours of neural crest origin, composed of mature ganglion cells and Schwann cells [1,2]. They most commonly arise in the posterior mediastinum and retroperitoneum, whereas cervical involvement is rare [3,4]. Although ganglioneuromas may occur in association with neurofibromatosis type 1 (NF1), this presentation has been infrequently reported [5].

Early and accurate diagnosis of cervical ganglioneuroma is clinically important because lesions in the carotid space are located close to major vessels and cranial and sympathetic nerves. However, distinguishing these lesions from other carotid space masses can be challenging because their clinical and radiological features may overlap with those of carotid body tumours, vagal schwannomas, sympathetic chain schwannomas, and other neurogenic tumours. Careful radiological assessment and multidisciplinary evaluation are therefore essential for surgical planning and for reducing the risk of neurovascular complications.

Surgical excision remains the treatment of choice and is generally associated with an excellent prognosis. Nevertheless, postoperative neurological complications may occur because of the close relationship between cervical ganglioneuromas and the sympathetic chain [4,6]. One such complication is Horner syndrome, which results from disruption of the cervical sympathetic pathway and is characterised by ipsilateral ptosis, pupillary constriction (miosis), and reduced facial sweating (anhidrosis). Depending on the degree of nerve injury, the syndrome may be temporary or permanent.

We report the case of a 24-year-old woman with sporadic NF1 who presented with a right-sided neck mass and discomfort. Magnetic resonance imaging demonstrated a well-defined fusiform lesion within the right carotid space, extending from C1 to C6 and causing displacement and splaying of the carotid bifurcation. This radiological appearance initially suggested a carotid body tumour [7-9]. Following multidisciplinary review, the mass was excised through a transcervical approach, and histopathological examination confirmed a ganglioneuroma with degenerative changes.

Postoperatively, the patient developed transient Horner syndrome, which resolved completely within six months. Follow-up imaging showed no evidence of recurrence. This case highlights the importance of considering cervical ganglioneuroma in the differential diagnosis of carotid space masses, particularly in patients with NF1, and of recognising the potential risk of temporary sympathetic dysfunction following surgical resection [3-6,8].

## Case presentation

A 24-year-old woman with a 10-year history of sporadic NF1 presented with right-sided neck pain and temporomandibular discomfort. She denied dysphagia, dysphonia, dyspnoea, constitutional symptoms, or other compressive symptoms. The diagnostic evaluation proceeded from clinical assessment to ultrasound and magnetic resonance imaging, followed by multidisciplinary review and surgical treatment. Postoperative clinical and radiological follow-up continued for four years.

Clinical examination demonstrated a firm, non-tender, non-pulsatile mass within the right anterior cervical triangle. Multiple cutaneous neurofibromas were present over the trunk and neck, consistent with NF1. Flexible nasoendoscopy showed preserved vocal cord mobility.

**Figure 1:**
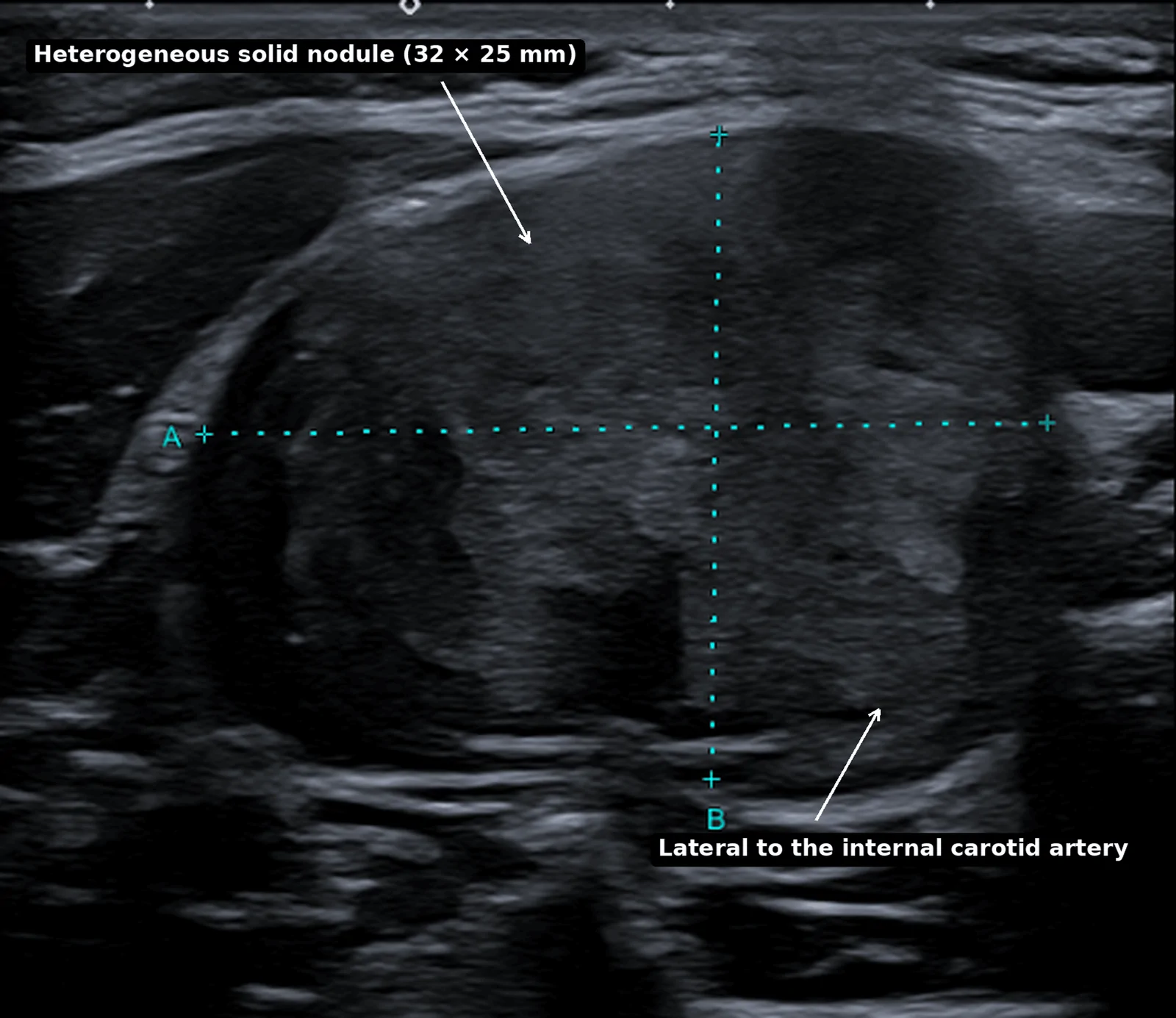
USG of focal heterogeneous solid nodule in the right neck lying just lateral to the internal carotid artery measuring 32 x 25 mm. Ultrasonographic image of the right neck demonstrating a well-defined heterogeneous solid nodule measuring 32 × 25 mm, situated lateral to the internal carotid artery, corresponding to the cervical mass identified on MRI.

**Figure 2:**
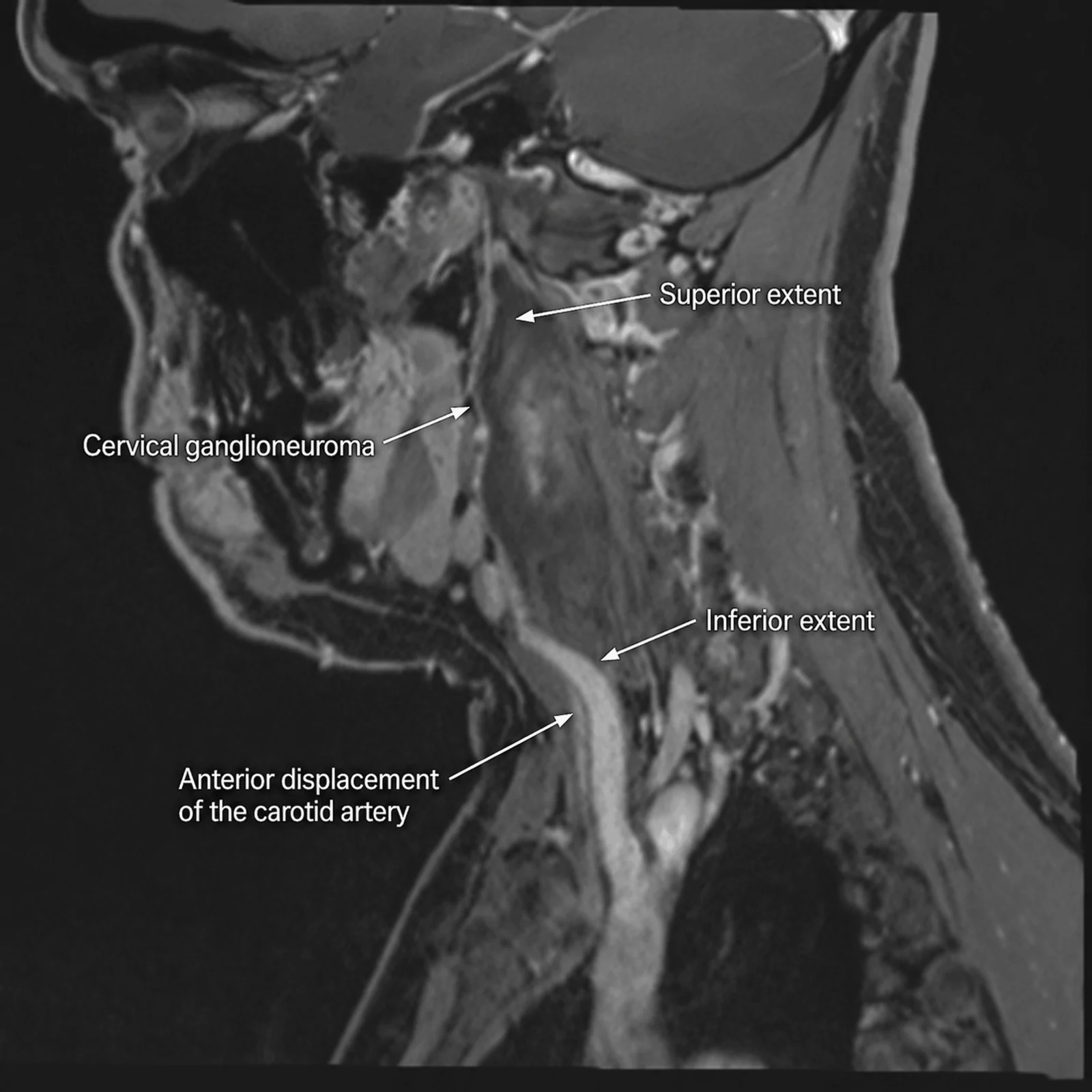
Sagittal contrast-enhanced T1-weighted MRI demonstrating a right carotid-space mass. Sagittal contrast-enhanced T1-weighted MRI of the neck demonstrates a large, sharply circumscribed fusiform mass within the right carotid space. The lesion extends from the level of the oropharynx at C1 to the glottis at C6 and measures 3.9 × 3.3 × 9.3 cm. Its elongated configuration and carotid-space location support a neurogenic origin. The arrow indicates the tumour.

**Figure 3:**
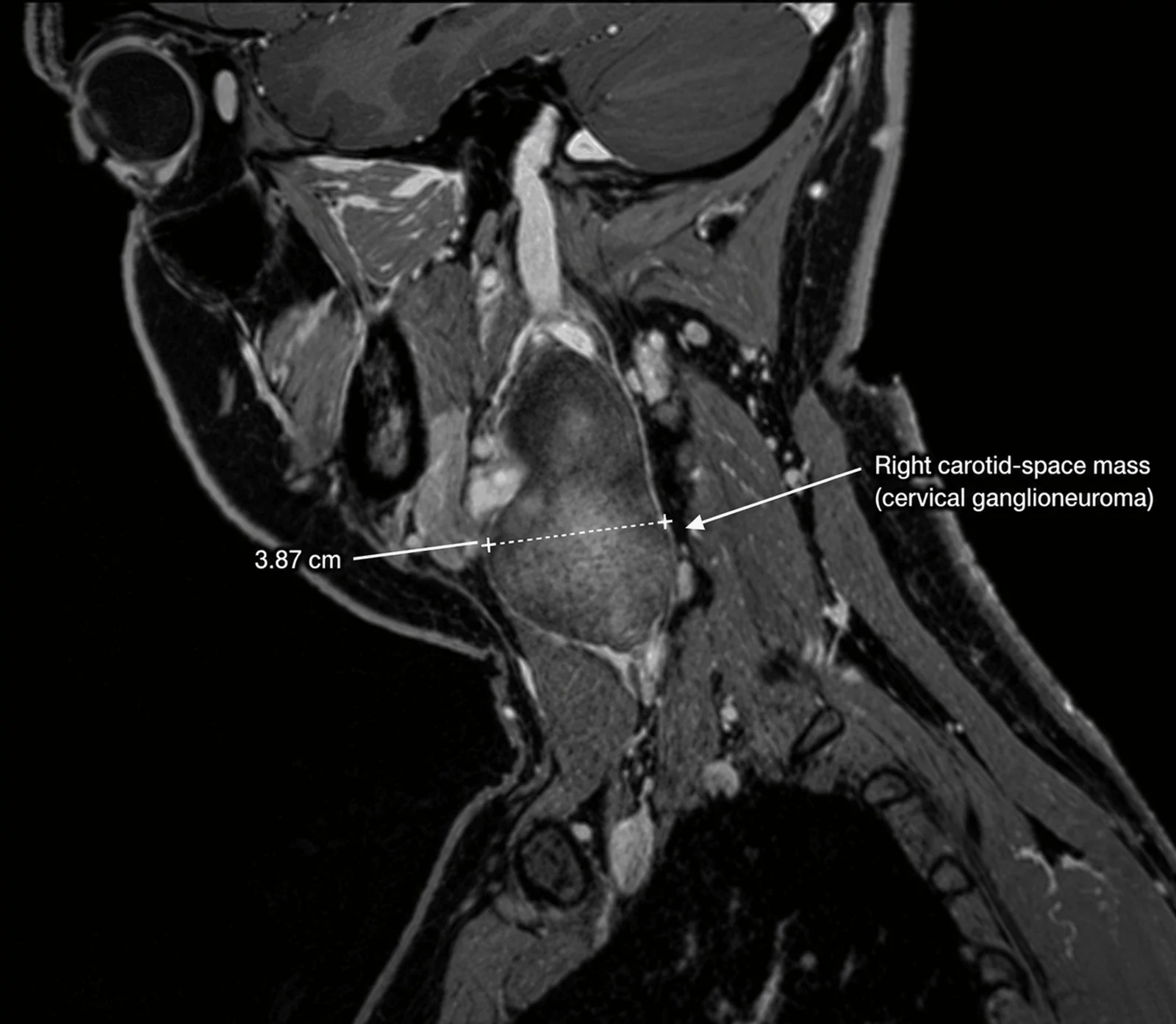
Sagittal T2-weighted MRI demonstrating a right carotid-space ganglioneuroma Sagittal T2-weighted MRI of the neck demonstrating a well-circumscribed right carotid-space mass measuring approximately 3.87 cm in maximal anteroposterior diameter. The lesion is predominantly T2 hyperintense and shows imaging features of a neurogenic tumour, including a target sign and a “nodule-in-nodule” appearance. Histopathological examination subsequently confirmed a cervical ganglioneuroma.

**Figure 4:**
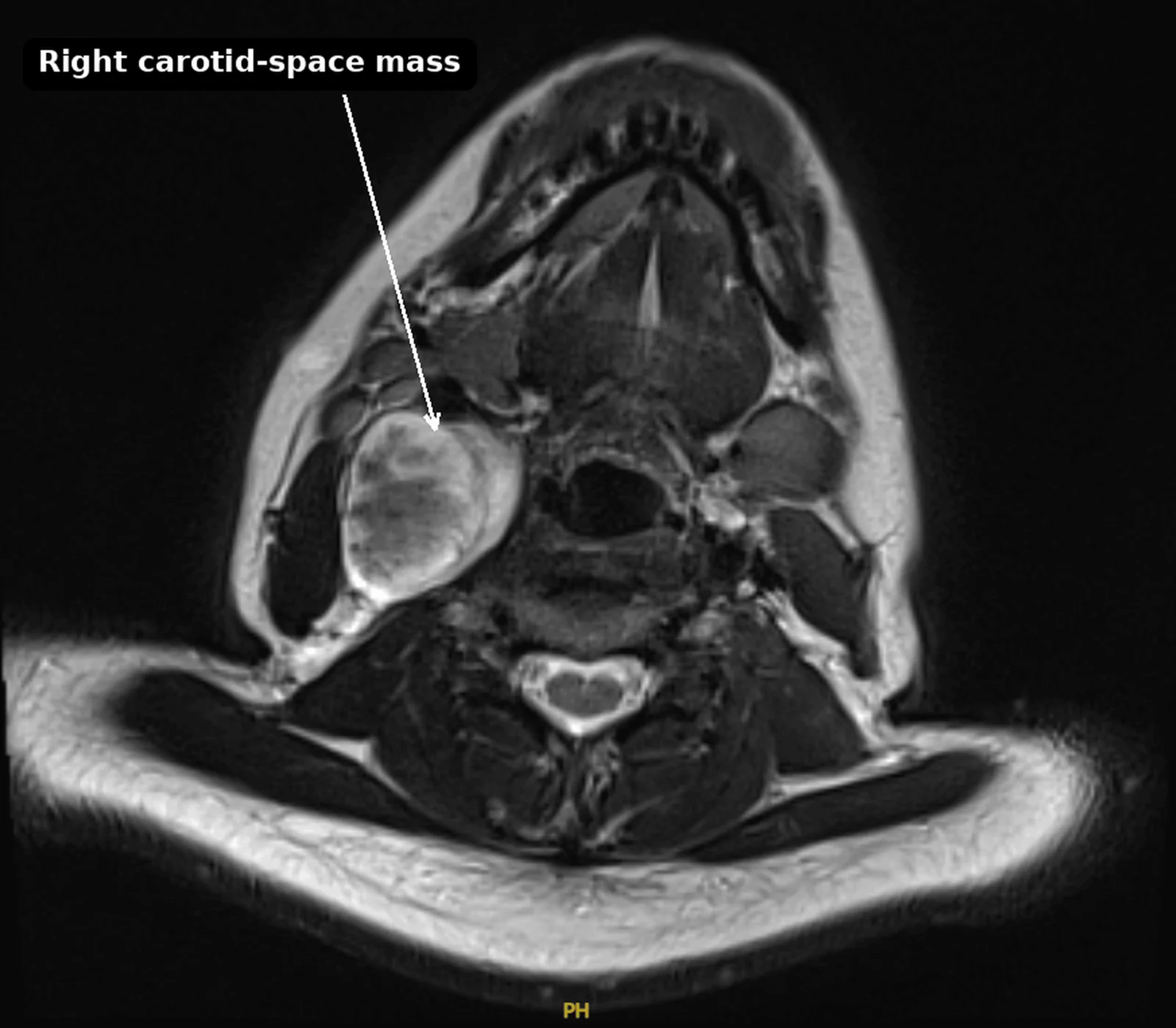
Axial T2-weighted MRI of the neck demonstrating a well-circumscribed right carotid-space mass with anterolateral displacement of the adjacent carotid vessels, supporting a neurogenic origin. Histopathological examination subsequently confirmed a cervical ganglioneuroma. Axial T2-weighted BLADE MRI (Siemens Healthineers, Erlangen, Germany) of the neck demonstrating a well-circumscribed right carotid-space mass with heterogeneous T2 hyperintensity and anterolateral displacement of the adjacent carotid vessels, supporting a neurogenic origin. Histopathological examination subsequently confirmed a cervical ganglioneuroma.

Ultrasound demonstrated a heterogeneous solid lesion measuring 32 × 25 mm lateral to the internal carotid artery (Figure 1). Magnetic resonance imaging revealed a well-circumscribed fusiform mass within the right carotid space, extending from C1 to C6 and measuring 3.9 × 3.3 × 9.3 cm (Figures 2, 3). The lesion displaced the common carotid artery anterolaterally and caused splaying of the carotid bifurcation. These findings were suggestive of a carotid space neurogenic tumour, although carotid bifurcation splaying may resemble the appearance of a carotid body tumour and complicate differentiation from paragangliomas and sympathetic chain lesions (Figure 4) [7-9]. A smaller contralateral lesion with similar radiological characteristics was also identified.

The differential diagnoses included vagal schwannoma, sympathetic chain tumour, neurofibroma, paraganglioma, and carotid body tumour [7-9]. Following multidisciplinary team discussion, surgical excision of the right-sided lesion was undertaken through a transcervical approach.

Intraoperatively, the tumour extended from the skull base to the level of the laryngeal framework and was closely related to the surrounding neurovascular structures. The vagus, accessory, and hypoglossal nerves were identified and preserved, and the major vascular structures remained intact. The lesion was excised en bloc.

Macroscopically, the excised specimen consisted of a soft tissue nodule measuring 65 × 30 × 35 mm, with nerve stumps identified at opposite poles. The cut surface was homogeneous, pale yellow, and myxoid, with no gross breach of the surrounding capsule.

Microscopically, the tumour was circumscribed by a thin capsule and was predominantly composed of bland spindle cells arranged haphazardly within a loose fibrous stroma. Scattered mature ganglion cells were present, particularly toward the periphery. Occasional enlarged hyperchromatic nuclei were identified, consistent with ancient-type atypia. There was no mitotic activity or evidence of malignancy in the multiple sections examined. The tumour focally touched the surgical margin.

At both poles of the lesion, prominent ganglion cells were observed within loose fibrous stroma adjacent to nerve tissue, supporting an origin from an associated nerve. The overall findings were consistent with ganglioneuroma, a Schwannian stroma-dominant neuroblastic tumour composed of Schwannian spindle cell stroma and mature ganglion cells without immature neuroblastic elements [1,2].

Five level II cervical lymph nodes were also examined. They demonstrated reactive changes, including prominent follicular hyperplasia and occasional sinus histiocytosis, with no evidence of malignancy. The final histopathological diagnosis was ganglioneuroma with ancient-type atypia, focally touching the surgical margin. The cervical lymph nodes showed reactive hyperplasia only.

Postoperatively, the patient developed ipsilateral ptosis and miosis, consistent with Horner syndrome. Ophthalmological assessment confirmed sympathetic dysfunction. Postoperative Horner syndrome is a recognised complication following resection of cervical ganglioneuromas and other cervical sympathetic-chain tumours [4,6]. Her symptoms gradually improved and resolved completely within six months.

Follow-up MRI performed four months after surgery showed no residual disease. Serial imaging over four years demonstrated no recurrence of the excised tumour and no progression of the smaller contralateral lesion.

## Discussion

Ganglioneuromas represent the fully differentiated end of the neuroblastic tumour spectrum and are composed of mature ganglion cells within Schwannian stroma [1,2]. They are slow-growing, biologically benign lesions that most commonly arise in the posterior mediastinum and retroperitoneum. Head and neck involvement is uncommon and accounts for only a small proportion of reported cases [3,4].

Cervical ganglioneuromas usually arise from the sympathetic chain and commonly present as painless, slowly enlarging neck masses [3,4]. On MRI, they are typically well circumscribed and displace rather than invade adjacent structures [8]. In this case, the elongated fusiform morphology, predominantly high T2 signal, target sign, mild patchy internal enhancement, and displacement of the carotid vessels supported a neurogenic origin. However, these findings were not specific enough to establish the exact tumour type preoperatively. The radiological differential diagnosis included neurofibroma, vagal schwannoma, sympathetic-chain tumour, paraganglioma, and carotid body tumour because these lesions may show overlapping appearances and vascular displacement patterns [7-9]. Histopathological examination was therefore essential and demonstrated mature ganglion cells within Schwannian stroma, confirming ganglioneuroma [1,2].

The current case shares several features with previously reported cervical ganglioneuromas, including a slow-growing carotid space mass, sympathetic chain localisation, displacement of nearby neurovascular structures, and a benign postoperative course [3,4]. Its extensive craniocaudal involvement from C1 to C6, association with NF1, bilateral neurogenic lesions, and transient postoperative Horner syndrome add to the relatively limited literature on cervical ganglioneuroma in this clinical setting. Although NF1 is strongly associated with peripheral nerve-sheath tumours, ganglioneuroma in patients with NF1 remains uncommon [5]. The additional contralateral neurogenic lesion and diffuse cutaneous neurofibromas in this patient are consistent with the underlying tumour predisposition associated with NF1.

Complete surgical excision is the preferred treatment and is generally curative [3,4]. Nevertheless, resection can be technically challenging because of the close relationship between cervical ganglioneuromas, the sympathetic chain, major vessels, and lower cranial nerves. Postoperative neurological deficits may therefore occur. Horner syndrome results from interruption of the cervical sympathetic pathway and is characterised by ipsilateral ptosis, miosis, and, in some patients, reduced facial sweating. It is a recognised complication after resection of cervical sympathetic-chain tumours and may result from traction, devascularisation, or direct injury to sympathetic fibres [4,6]. In the present case, the symptoms were transient and resolved completely within six months, suggesting temporary rather than permanent sympathetic dysfunction.

Long-term outcome data for cervical ganglioneuroma remain limited. Our patient has remained free of recurrence for four years following surgery, while the smaller contralateral lesion has remained stable. This favourable course is consistent with the benign biological behaviour and good prognosis generally reported after complete excision [3,4]. Continued surveillance remains appropriate, particularly in patients with NF1 and additional neurogenic lesions.

## Conclusions

Cervical ganglioneuroma is a rare cause of a carotid space mass, including in patients with NF1, and may closely mimic other neurogenic tumours on imaging. Although complete surgical excision is usually curative, this case highlights the risk of postoperative Horner syndrome because of the tumour’s close anatomical relationship with the cervical sympathetic chain. Multidisciplinary evaluation is important for accurate radiological assessment, surgical planning, and counselling regarding potential neurological complications. Long-term clinical and radiological follow-up is also advisable, particularly in patients with NF1 or additional neurogenic lesions.

## References

[1] Shimada H, Ambros IM, Dehner LP, Hata J, Joshi VV, Roald B (1999). Terminology and morphologic criteria of neuroblastic tumors: recommendations by the International Neuroblastoma Pathology Committee. Cancer.

[2] Okamatsu C, London WB, Naranjo A (2009). Clinicopathological characteristics of ganglioneuroma and ganglioneuroblastoma: a report from the CCG and COG. Pediatr Blood Cancer.

[3] Ma J, Liang L, Liu H (2012). Multiple cervical ganglioneuroma: a case report and review of the literature. Oncol Lett.

[4] Saadat S, Fritz C, Tran D (2022). A systematic review of cervical ganglioneuromas. OTO Open.

[5] Wang C, Niu D, Zhai N (2024). Cervical ganglioneuroma associated with neurofibromatosis type 1. J Craniofac Surg.

[6] Lima AF, Moreira FC, Menezes A, Dias L (2018). Cervical ganglioneuroma in pediatric age: a case report. Turk Arch Otorhinolaryngol.

[7] Britten AG, Entezami P, Chang BA (2020). Cervical ganglioneuroma mimicking a carotid body tumour. BMJ Case Rep.

[8] Chen Z, Wei Y, Lu C (2025). CT and MRI imaging of mass in the carotid space: a pictorial review. Quant Imaging Med Surg.

[9] Wang CP, Hsiao JK, Ko JY (2004). Splaying of the carotid bifurcation caused by a cervical sympathetic chain schwannoma. Ann Otol Rhinol Laryngol.

